# The Importance of the Immune System and Molecular Cell Signaling Pathways in the Pathogenesis and Progression of Lung Cancer

**DOI:** 10.3390/ijms24021506

**Published:** 2023-01-12

**Authors:** Jolanta Smok-Kalwat, Paulina Mertowska, Sebastian Mertowski, Konrad Smolak, Aleksandra Kozińska, Filip Koszałka, Wojciech Kwaśniewski, Ewelina Grywalska, Stanisław Góźdź

**Affiliations:** 1 Department of Clinical Oncology, Holy Cross Cancer Centre, 3 Artwinskiego Street, 25-734 Kielce, Poland; 2 Department of Experimental Immunology, Medical University of Lublin, 4a Chodzki Street, 20-093 Lublin, Poland; 3 Student Research Group of Experimental Immunology, Medical University of Lublin, 4a Chodzki Street, 20-093 Lublin, Poland; 4 Department of Gynecologic Oncology and Gynecology, Medical University of Lublin, 20-081 Lublin, Poland; 5 Institute of Medical Science, Collegium Medicum, Jan Kochanowski University of Kielce, IX Wieków Kielc 19A, 25-317 Kielce, Poland

**Keywords:** immunopathogenesis, lung cancer, genetic association, signal transduction pathway, immune system

## Abstract

Lung cancer is a disease that in recent years has become one of the greatest threats to modern society. Every year there are more and more new cases and the percentage of deaths caused by this type of cancer increases. Despite many studies, scientists are still looking for answers regarding the mechanisms of lung cancer development and progression, with particular emphasis on the role of the immune system. The aim of this literature review was to present the importance of disorders of the immune system and the accompanying changes at the level of cell signaling in the pathogenesis of lung cancer. The collected results showed that in the process of immunopathogenesis of almost all subtypes of lung cancer, changes in the tumor microenvironment, deregulation of immune checkpoints and abnormalities in cell signaling pathways are involved, which contribute to the multistage and multifaceted carcinogenesis of this type of cancer. We, therefore, suggest that in future studies, researchers should focus on a detailed analysis of tumor microenvironmental immune checkpoints, and to validate their validity, perform genetic polymorphism analyses in a wide range of patients and healthy individuals to determine the genetic susceptibility to lung cancer development. In addition, further research related to the analysis of the tumor microenvironment; immune system disorders, with a particular emphasis on immunological checkpoints and genetic differences may contribute to the development of new personalized therapies that improve the prognosis of patients.

## 1. Introduction

**Figure 1:**
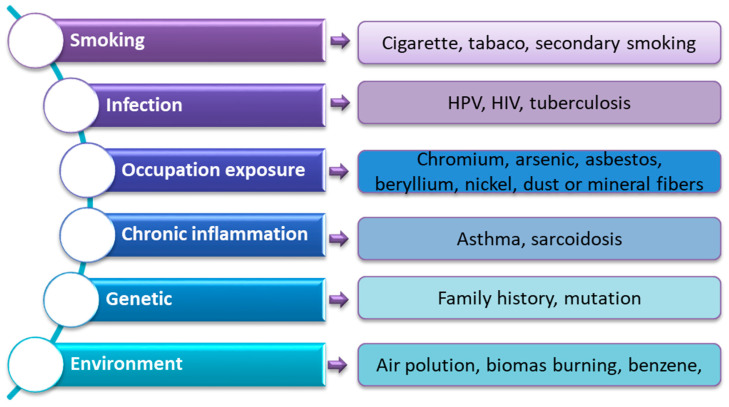
Risk factors for lung cancer (based on [4,5,6,7,8,9,10,11]).

Lung cancer is one of the diseases with the highest mortality rate in the world. According to the World Health Organization, in 2020 the number of deaths caused by this cancer amounted to 1.80 million people, which accounted for 25% of the recorded deaths due to cancer [1,2]. The American Cancer Society lung cancer statistics for 2022 show that approximately 236,740 new cases of lung cancer were diagnosed in the United States alone (117,910 in men and 118,830 in women) and approximately 130,180 people died from lung cancer (68,820 men and 61,360 women) [3]. In addition to gender and age differences (diagnosed patients are >65 years of age), many environmental, genetic and lifestyle factors are involved in the development of lung cancer (Figure 1) [4,5,6,7,8,9,10,11]. However, more and more scientific studies highlight the extremely important role of genetic changes in the development and progression of this type of cancer [12,13,14,15].

Genetic aberrations and epigenetic changes occurring in the human body have a significant impact on proto-oncogenes (genes that stimulate cell growth, which may be transformed into oncogenes as a result of mutation) and suppressor genes (genes encoding proteins, whose task is to inhibit cell growth and differentiation and to maintain cell stability; mutations in these genes lead to uncontrolled cell proliferation), facilitating the development and migration of cancer cells. However, this is only one aspect of the process [16,17,18,19,20]. Under conditions of homeostasis, the immune system has an extremely important potential for monitoring, recognizing and destroying cancer cells. However, these cells have developed several mechanisms that allow them to escape the surveillance of the immune system [21,22,23,24]. Therefore, all deregulations in the human body related to the functioning of the immune system should be considered as the second potential process involved in the pathogenesis of cancer, including lung cancer [25].

Despite many studies on the risk factors, classification and pathogenesis of lung cancer, very little data are devoted to disorders of the immune system and signal transduction pathways, which, as a result of genetic changes, determine the development of cancer cells. The aim of this publication is to review the literature on the importance of disorders of the immune system and the accompanying molecular implications in the pathogenesis of lung cancer. We paid particular attention to the importance of cancer antigens and neoantigens, as well as the role of immune checkpoints and abnormalities in cell signaling pathways that contribute to the multifaceted and multistage carcinogenesis of lung cancer.

## 2. Results and Discussions

### 2.1. Molecular and Histological Classification of Lung Cancer

**Figure 2:**
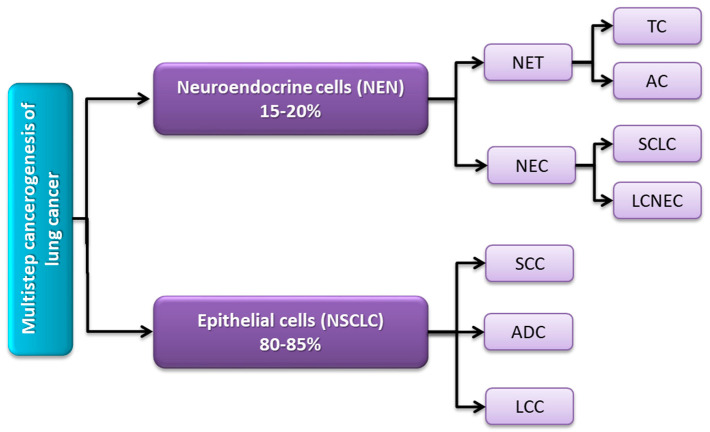
Classification of different subtypes of lung cancer (based on [30,31,32]).

The data from the literature classified lung cancer histologically into two main types: neuroendocrine cell-derived small-cell lung cancer (SCLC), which accounts for 15–20% of all lung cancer patients, and epithelial cell-derived non-small-cell lung cancer (NSCLC), which accounts for 80–85% [19,26,27,28]. Neuroendocrine neoplasms (NENs) of the lung include two characteristic subtypes: neuroendocrine tumors (NETs), consisting of typical (TCs) and atypical (ACs) carcinoids; and full-fledged carcinoids (NECs), which include large-cell neuroendocrine carcinomas (LCNECs) and small cell carcinomas (SCLCs). Detailed studies resulted in the morphological and molecular characterization of these subtypes (Figure 2) [28,29].

In the case of NSCLC lung cancer, three main histological subtypes can be distinguished: adenocarcinoma (ADC), accounting for about 40% of all lung cases; squamous cell carcinoma (SCC), which is strongly associated with a smoking history and accounted for about 20% of all cases lung cancer; and large cell carcinoma (LCC). As indicated by histological studies, lung cancer is characterized by high heterogeneity, which is also reflected at the molecular level. Thanks to the development of genomic research, it was possible to determine the molecular patterns of each of the subtypes of lung cancer, which, according to research data, also differed depending on the histological subtype [33,34,35,36].

The TSG analysis for selected lung cancer subtypes based on the COSMIC Cancer Gene Census showed that there are certain genes in which mutations occur much more often. We can indicate both genes that are not specific to a specific subtype, such as EGFR or TP53, as well as those that are characteristic of a specific subtype of lung cancer (Table 1).

Furthermore, clinical studies have shown that molecularly defined lung cancer subgroups may be correlated with characteristics such as ethnicity [38,39,40], smoking history [41,42,43], treatment sensitivity [44,45,46] or prognosis [47].

Most of the genes indicated in the literature and databases encode proteins involved in the signal transduction process (kinases, transcription factors, receptors), which, as a result of mutations, introduce disturbances in the signal transduction pathway between cells.

Specific proteins could be used for differential diagnosis, facilitating the diagnosis of a specific type of lung cancer. Examples of proteins that have been tested and assessed for differential diagnosis include p63 in correlation with the TTF-1 transcription factor [48]. MUC5B was also correlated with the transcription factor TTF-1 [49]. The first protein was studied in the context of small-cell lung cancer compared to poorly differentiated non-keratinizing squamous cell carcinoma [48]. In these studies, the researchers demonstrated the utility of evaluating a panel of p63 and TTF-1 antibodies as useful markers in distinguishing SCLC from poorly differentiated non-keratinizing SCC on surgical, biopsy and cytology sections. The researchers based their conclusions on the positive expression of the p63 protein, in the negative correlation of the reaction for TTF-1, shown in subjects with differentiated non-keratinizing SCC, while the opposite results were obtained for SCLC. Similar conclusions were obtained in studies conducted by Veena et al., and in the summary of their research the researchers indicated that obtaining positive results for TTF1 and negative results for p63 can help in the diagnosis of adenocarcinoma, while samples that are positive for p63 and negative for TTF1 negative can be indicated and help in the diagnosis of squamous cell carcinoma [50]. In turn, another study conducted by Nagashio et al. indicated the usefulness of MUC5B protein determination in correlation with TTF-1, which may be useful for differential diagnosis between AC and SCC. In addition, it has been suggested that an assessment of the expression of the above factors could be a prognostic indicator for the prognosis of patients with pulmonary AC [49]. This means that any abnormalities of these genetic factors or disorders in the functioning of the pathway itself should be carefully analyzed in terms of their involvement in the development and progression of lung cancer.

### 2.2. Disorders of Cell Signaling Pathways as an Element of Lung Cancer Development and Progression

The development of new research techniques in the field of genomic and proteomic research has contributed to the increased interest of many researchers in the interactions between cancer cells, their unique microenvironment and intercellular communication pathways [51]. Analyses have been launched to demonstrate the occurrence of genetic aberrations and epigenetic changes that may determine an increased risk of lung cancer, but also affect the selection of therapeutic therapies and their therapeutic success. In recent years, several papers on this issue have appeared in the literature, which have allowed the classification of the observed disorders into three characteristic pathways, which, as the researchers indicated, can direct cells towards a malignant phenotype. These include signal transduction pathways that stimulate cell growth, pathways associated with tumor suppressor genes and pathways associated with cell evasion of apoptosis [52,53,54]. Detailed characteristics of these three groups of pathways with the most important genes/proteins involved in the development and progression of lung cancer are presented in Table 2.

### 2.3. Epigenetic Changes Involved in the Pathogenesis of Lung Cancer

All the cells of the human body have almost identical genetic information, yet the cells of different tissues are very different from each other. This remarkable diversity is due to the different use of the same genetic information through epigenetic mechanisms. These mechanisms allow the emergence of some kind of permanent gene expression patterns for a given differentiated cell, with a simultaneous short-term and reversible change in their expression. Research on the epigenome (i.e., a set of DNA and histone protein modifications) developed in recent years has led to the determination of a number of molecular mechanisms that are epigenetic modifications in the course of lung cancer [106,107,108]. Such mechanisms include the following:

(1) A change in DNA methylation status within the CpG islands of tumor suppressor genes;
(2) Covalent modifications of histone tails;
(3) Regulation of genes by micro-RNAs (miRNAs).

The first mechanism can be considered in two ways: in chemical terms (the process in which the methyl group -CH_3_ is transferred between two molecules) and in biological terms (i.e., the effect of methylation on the functioning of DNA). In response to various external factors, the DNA of each person undergoes changes that are designed to adapt the functioning of the body to changing conditions [109]. Therefore, the correct course of this process plays a key role for the efficient functioning of cells and organs because it determines the expression and activity of individual genes. As a result of disorders of methylation pathways in the human body, there may be abnormalities related to immune reactions or the development of cancer (genetically determined or resulting from spontaneous mutations) [110,111]. Thus, methylation has become the subject of many intensive studies aimed at elucidating its detailed role in the pathogenesis of neoplastic diseases. According to the available literature, three types of abnormal methylation may occur in the development of this type of disease: global hypomethylation, hypermethylation of suppressor genes, and regulation of miRNA activity by methylation [112,113]. Hypomethylation is a process observed in the course of many diseases, especially those of a chronic nature, such as autoimmune diseases and cancer. According to some scientists, this process can reduce the stability of chromosomes and activate proto-oncogenes, and, in non-promoter regions of genes, it can weaken the stability of the genome by demethylating transposons. The phenomenon of global hypomethylation does not depend on local changes in DNA methylation in regulatory regions of genes. Genetic and cellular data support the idea that global DNA methylation is responsible for genome integrity and can also lead to chromosomal aberrations [114,115,116].

Hypermethylation, i.e., an increased degree of methylation, causes silencing of DNA repair genes and tumor suppressor genes (anti-oncogenes). The result of this process is that there is no limit to cell proliferation and there is a high probability that the abnormal cell will not be directed to the apoptotic pathway. In addition, the hypermethylation of DNA repair genes turns off genes that code for proteins responsible for repairing abnormal genes [117]. Since hypermethylation of promoter regions results in a loss of gene function, it has a major impact on cell functioning and can inactivate genes and key biological pathways [118]. In the course of neoplastic diseases, we can observe both the loss of methylation in non-coding regions of the genome, which in turn leads to disturbances in its stability, or the methylation of CpG islands in the tumors themselves, which results in a loss of expression of tumor suppressor genes. In lung cancer, studies have indicated that many genes have been silenced by methylation, including RARB (retinoic acid receptor beta), CDKN2A (cyclin dependent kinase inhibitor 2A), TIMP3 (TIMP metallopeptidase inhibitor 3), MGMT (methylguanine methyltransferase), DAPK (death associated protein kinase 1), CDH13 (cadherin 13) and CDH1 (cadherin 1) [119,120,121,122,123,124,125,126,127,128,129,130]. Researchers suggested that restoring the expression of silenced genes in the course of lung cancer may be a new therapeutic target.

The second mechanism of epigenetic changes involves modifications of histones and their implementing enzymes in response to internal and external stimuli [131]. Disturbances in their functioning can lead to many unfavorable changes, such as chromatin compaction, changes in nucleosome dynamics and abnormalities at the level of transcription. All changes contribute to the imbalance of gene expression, which in the case of the development of cancer, is observed as a gain or loss of function, overexpression, suppression by promoter hypermethylation, chromosome translocation or mutations of histone-modifying enzymes/complexes and even histone modification sites [132]. In lung cancer, scientists have observed a specific combination of histone markers resulting from epigenetic changes that include the deacetylation of H3 and H4 histones, loss of lysine 4 trimethylation of histone H3 and an increase in the trimethylation of H3K9 and H3K27. In 2007, Barlési et al. showed that the acetylation and trimethylation states of H2 and H3 can be a kind of prognostic marker in the course of NSCLC. They showed that the predictive value of epigenetic changes involved many histones, and of particular note were H2A (H2AK5ac) and H3 (H3K4me2, H3K9ac), which were higher in the early development of NSCLC [133].

The team of Van Den Broeck et al. showed in 2008 that there is increased H4K5/H4K8 acetylation as well as a loss of H4K20 trimethylation in NSCLC and pre-invasive bronchial dysplastic lesions. In addition, they found that the loss of H4K20 trimethylation was significantly associated with the early stage I ADC subpopulation, and their data indicated shortened survival [134]. Similar conclusions were also drawn by researchers from the team of Seligson et al. in 2009, who showed that the cellular levels of H3K4me2 and H3K18ac were relatively low, and the identified histone modification trends were independent predictors of prognosis in the course of ADC [135]. Studies presented in the literature suggest that reduced cellular levels of specific histone modifications may be a kind of predictive factor related to the prognosis of patients with lung cancer.

The third mechanism of epigenetic changes concerns the regulation of miRNAs. This is a family of small non-coding RNAs (21–25 nucleotides long) that can negatively affect the translation of messenger RNA (mRNA) and promote its degradation by base pairing with complementary mRNA target sites [135]. Thanks to the development of such a mechanism, miRNAs can change gene expression after the transcription process. The literature describes over 500 miRNAs found in humans, which can function both as oncogenes, but also as tumor suppressor genes [136]. As a result of intensive research in recent years, scientists have managed to classify some miRNAs involved in the pathogenesis of lung cancer into eight groups related to signaling pathways and cellular processes: sustaining proliferative signaling; evading growth suppressors; enabling replicative immortality; activating invasion and metastasis; inducing angiogenesis; deregulating circular energetics; resisting cell death; avoiding immune destruction and tumors promoting inflammation. Detailed data are presented in Table 3. The scientists indicated that the use of miRNAs in the future may allow the identification of histological subtypes of lung cancer, which will increase the diagnostic possibilities and their participation as molecular biomarkers in screening tests, as well as determine whether to distinguish primary from metastatic tumors [137]. In addition, researchers hope that miRNAs will play an important role in determining patient prognoses and tailoring personalized therapies for lung cancer in the future [138].

### 2.4. The Importance of the Immune System in the Pathogenesis of Lung Cancer

**Figure 3:**
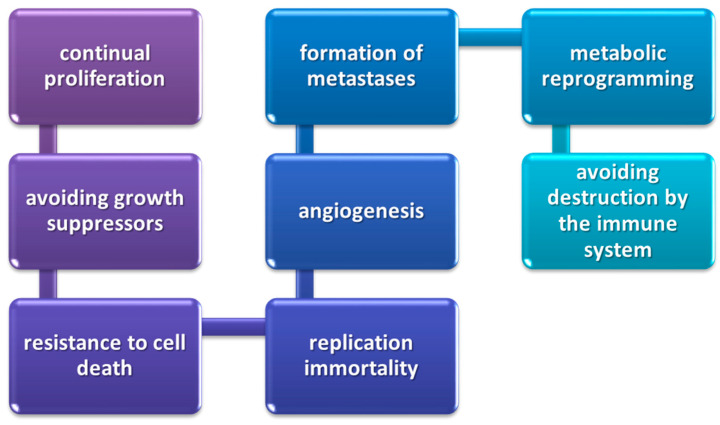
Stages of cancerogenesis development and progression (based on [300]).

In the condition of immune homeostasis, monitoring, recognition and destruction of foreign antigens and cancer cells take place [299]. The latter group is capable of an uncontrolled proliferation and spread throughout the human body, as well as attacking healthy tissues or cells. Tumor development and progression is a multistage process and involves eight characteristic processes that are also observed in the development of lung cancer (Figure 3) [300].

Understanding the role of the immune system in the development and progression of many types of cancer has become one of the most intensive research objects of recent decades.

Based on this type of research, cancer immunotherapies are being developed that target the immune microenvironment of tumors. Some researchers have indicated that type 1 and type 2 immune responses are important in the progression of cancer. Type 1 immune responses are considered important in fighting cancer, and immunotherapies are aimed at increasing the activity of this type of immune response [301]. Such a conclusion seems correct because in NSCLC tumors Stankovic’s team identified major different subpopulations of T cells, and the second most common type of disease cell in NSCLC tumors was B cells [302].

Still, there are many cases of research in which the scientists have been able to discover only a small part or aspect of how the immune response works in fighting cancer cells.

That is why in order to better understand the interactions between cancer cells and the immune system, researchers looked at the contributions of components of the adaptive and innate immune responses, the involvement of tumor antigens, the importance of the tumor microenvironment and immune checkpoints [25,53].

#### 2.4.1. Importance of Tumor Antigens and Neoantigens

Immune surveillance assumes that cancer cells appear in the human body more often than the clinical manifestation of cancer, but their development is controlled by appropriate immune mechanisms that allow their detection and destruction [303,304]. This process is carried out by both the innate and adaptive immune systems and is assisted by cancer-specific antigens (tumor markers) that can be recognized by the immune system. Tumor markers are specific substances, often proteins, that are produced by the cancer tissue itself or sometimes by the body in response to the appearance of cancer. The presence or elevated levels of tumor markers in the blood or urine can help detect certain types of cancer, including lung cancer [305]. One group of antigens is known as tumor-associated antigens (TAAs), which are antigens that are overexpressed in cancer cells but can also be found in normal tissues, while the other group is tumor-associated antigens (TSAs). Both groups of antigens have become the subject of many studies aimed at supporting the diagnostic process and therapy of patients with cancer. In the case of lung cancer, the majority of the literature has focused on the search for tumor antigens that may be specific biomarker molecules in the course of NSCLC.

Research conducted by the team of Gure et al. in 2005 aimed to determine the level of expression of nine tumor antigens (NY-ESO-1, LAGE-1, MAGE-A1, MAGE-A3, MAGE-A4, MAGE-A10, CT7/MAGE-C1, SSX2 and SSX4) in over 500 patients diagnosed with NSCLC. The researchers showed that the expression of cancer genes in the testis, both cumulative and single, showed a significant relationship with the clinical characteristics of patients, i.e., with male sex, smoking history, cancer stage or the presence of metastases. In addition, the scientists’ Cox regression analysis showed that the expression of NY-ESO-1 and MAGE-A3 was correlated with a poor patient prognosis [306]. Another study carried out by Grunwald et al. in 2006 showed that the expression of seven genes (MAGE-A3, NY-ESO-1, LAGE-1, BRDT, HOM-TES-85, TPX-1 and LDHC) was associated with the development of NSCLC. Researchers have shown that 81% of NSCLC patients tested expressed at least one of the genes tested and 50% of the sample at least two of the genes. Additionally, the researchers showed that all but one of the genes are regulated by genomic methylation, and not all of them are co-expressed [307]. In 2020, the team of Palat et al. conducted research in which 12 selected cancer antigens were determined (CEA, MAGE-A1, MAGE-A3, MAGE-A4, PRAME, hTERT, HER2, MUC1, Survivin, STEAP1, SOX2 and NY-ESO-1), and their involvement in the pathogenesis of lung cancer was studied, with a particular emphasis on NSCLC subtypes [308]. Based on the analyses carried out, the researchers showed a higher expression of TAA SCCs in relation to ACs. In addition, they showed that the patients’ T-cell response to stimulation was significantly lower in patients with SCCs than those with ACs. Researchers have suggested that there are specific differences in T-cell function between NSCLC subtypes [308]. Some researchers have pointed out that cancer proteins circulating in serum or plasma, released from cancer cells, are few and their sensitivity, especially in terms of diagnostics, is low, especially in the early stages of cancer. Therefore, in recent years, scientists have used the fact that TAs are capable of inducing an immune response and stimulating the formation of autoantibodies associated with them (TAAb). They are created not only before, but also during the formation of the cancer itself, which means that they can be potential biomarker molecules that will be involved in the diagnostic process [309]. The researchers pointed out that TAAbs have several significant advantages that outweigh their use over TAs: the response of TAAbs to TAs is often enhanced by immune responses, allowing them to be more easily detected; TAAbs are relatively stable in body fluids, unlike TAs, and, therefore, according to the researchers, they are more stable; and TAAbs are highly specific and easily detectable in small sample volumes [310,311,312].

**Figure 4:**
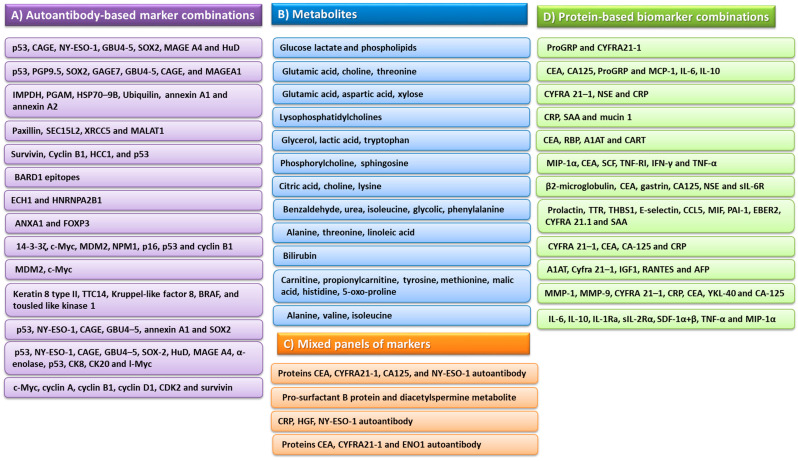
Biomarker molecules for use in the diagnostic process of lung cancer (based on [313,314,315,316,317,318,319,320,321,322,323,324,325,326,327,328,329,330,331,332,333,334,335,336]). Abbreviations: p53—tumor protein P53; CAGE—cancer-associated gene protein; NY-ESO-1—New York esophageal squamous cell carcinoma-1; SOX2—SRY-box transcription factor 2; MAGEA4—melanoma-associated antigen 4; HuD—ELAV-like protein 4; PGP 9.5—protein gene product 9.5; GAGE7—G antigen 7; MAGEA1—melanoma-associated antigen 1; IMPDH1—inosine monophosphate dehydrogenase 1; PGAM—phosphoglycerate mutase; HSP-9B—heat shock protein family A member 9B; SEC15L2—EXOC1 exocyst complex component 1; XRCC5—X-ray repair cross complementing 5; MALAT1—metastasis-related lung adenocarcinoma transcript 1; HCC1—nuclear protein Hcc-1; BARD1—BRCA1-associated RING domain protein 1; ECH1—Enoyl-CoA hydratase 1; HNRNPA2B1—heterogeneous nuclear ribonucleoprotein A2/B1; ANXA1—annexin A1; FOXP3—forkhead box protein P3; c-MYC—MYC proto-oncogene; MDM2—mouse double minute 2 homolog; NPM1—nucleophosmin 1; p16—cyclin-dependent kinase inhibitor 2A; TTC14—tetratricopeptide repeat domain 14; BRAF—serine/threonine-protein kinase B-raf; CK8—cytokeratin 8; CK20—cytokeratin 20; CDK2—cyclin-dependent kinase 2; CEA—carcinoembryonic antigen; CYFRA 21-1—cytokeratin 19 fragments; CA 125—cancer antigen 125; CRP—C-reactive protein; HGF—hepatocyte growth factor; ENO1—alpha-enolase; ProGRP—progastrin-releasing peptide; MCP1—monocyte chemoattractant protein 1; IL-6—interleukin-6; IL-10—interleukin-10; NSE—neuron-specific enolase; SAA—serum amyloid A; RBP—retinol-binding proteins; A1AT—alpha-1 antitrypsin; CART—cocaine and amphetamine-regulated transcript; MIP-1α—macrophage inflammatory proteins alpha 1; SCF—Skp, Cullin, F-box-containing complex; TNF RI—tumor necrosis factor receptor I; IFN-γ—interferon gamma; TNF-α—tumor necrosis factor alpha; sIL-6R—soluble interleukin-6 receptor; TTR—transthyretin; THBS1—thrombospondin-1; CCL5—CC motif chemokine ligand 5; MIF—macrophage migration inhibitory factor; PAI-1—plasminogen activator inhibitor 1; ERBB2—erb-b2 receptor tyrosine kinase 2; IGF1—insulin-like growth factor 1; RANTES—regulated upon activation, normal T-cell expressed and secreted; AFP—alpha fetoprotein; MMP-1—matrix metallopeptidase 1; MMP-9—matrix metallopeptidase 9; YKL-40—chitinase-3-like protein; IL-1Ra—interleukin 1 receptor antagonist; sIL-2Ra—human soluble interleukin 2 receptor alpha; SDF-1α+β—stromal cell-derived factor 1 alfa + beta.

More and more attention has been paid by scientists to the development of combinations of biomarker molecules found in the blood or sputum of lung cancer patients that could potentially contribute to the early detection of neoplastic changes. According to the data available from the literature, these molecules are usually associated in several combinations and classified into one of four groups: autoantibody-based marker combinations, metabolites, protein-based biomarker combinations and mixed panels of markers (Figure 4) [313,314,315,316,317,318,319,320,321,322,323,324,325,326,327,328,329,330,331,332,333,334,335,336].

Lung cancers carry a high mutational load (point mutations, misfolding, overexpression, abnormal glycosylation, shortening or abnormal degradation), which leads to the formation of unique peptides capable of inducing an immune response in the body, i.e., neoantigens. The formation of abnormal proteins as a result of their mutations can lead to their recognition by the host’s immune system, more specifically by the histocompatibility system (MHC) class I (MHC-I) or MHC class II. Neoantigens in the tumor cytosol are presented on MHC-I and recognized by CD8+ cytotoxic T cells, while neoantigens released into the tumor microenvironment can be taken up by circulating antigen-presenting cells [337,338]. In the literature, we can find studies indicating that neoantigens are specific to the patient and not to the tumor itself, which, according to the researchers, suggests that tumors avoid destruction due to the immune system. These properties make neoantigens increasingly recognized as key mediators of tumor-specific immune activation and they have been identified as potential targets for personalized cancer therapies (including cancer vaccines) [312,337,338,339]. Thanks to the development of molecular biology techniques, including next-generation sequencing, and the participation of bioinformatics, it is possible to use data to predict neoantigens, most often on the basis of their affinity to MHC-I. However, research in recent years has resulted in a much broader approach to the detection of neoantigens in lung cancer (especially NSCLC), taking into account both the stability of binding to MHC-I, as well as the level of gene expression or aggretopicity [340,341,342,343]. According to the literature, the median number of predicted neoantigens in NSCLC is quite variable. In 2016, Karasaki et al. conducted research aimed at identifying individual and specific somatic mutations occurring in NSCLC, which could be a new method of neoantigen-based immunotherapy. The researchers found from 13 to 659 neoantigens for ADC (median 46) and from 10 to 145 neoantigens for SCC (median 95.5) [344]. Other reports in the literature state that the number of neoantigens detected in NSCLC ranged from 63 to 214 neoantigens per tumor. Researchers have observed that increased numbers of neoantigens were noted among patients who had molecular signatures associated with cigarette smoking and increased expression of programmed death ligand 1 (PD-L1) [339,345,346].

#### 2.4.2. Importance of Immune Checkpoints

**Figure 5:**
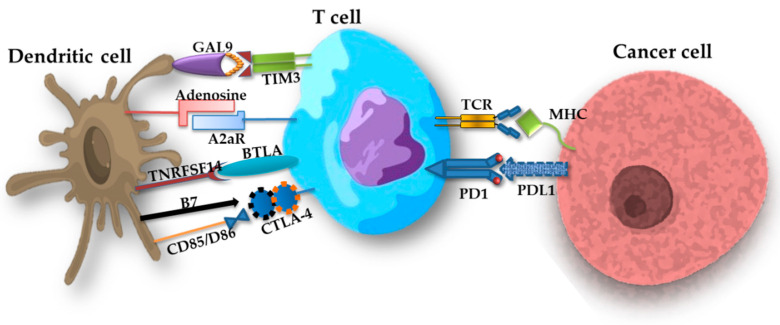
Exemplary interactions between a T lymphocyte and a tumor cell and dendritic cell, including immunological checkpoints (based on [350,351,352,353,354,355]).

Immune checkpoints are systems of protein receptors and ligands exposed on the surface of cells of the immune system (especially T lymphocytes) that modulate the body’s immune responses [347,348,349]. Many studies have shown that they are involved in altering the ability of the immune system to recognize malignant cells. In conditions of immune homeostasis, immune checkpoints provide self-tolerance by maintaining T lymphocyte activation, which causes inhibition of the immune response, but in the case of cancer, they are inhibited and the immune system is reactivated so that it is able to launch an attack on cancer cells [350,351]. In the literature, we found data on the detailed mechanisms of action of almost 20 significant modulators of immune checkpoints (Table 4), such as LAG-3, TIM3 and TIGIT, as well as the programmed cell death receptor 1 (PD-1) and a protein associated with cytotoxic T-4 lymphocytes (CTLA-4) (Figure 5), which have found their greatest application in clinical practice, including the treatment of lung cancer [350,351,352,353,354,355].

##### PD-1/PD-L1 Pathway

The PD-1 molecule together with its PDL-1/PD-L2 ligands as immune checkpoint molecules are responsible for suppressing the effector phase of activated T lymphocytes, reducing the inflammatory response and, consequently, autoimmunity [356]. These molecules can exist in the form of being bound to the cell membrane and soluble in serum and blood plasma [357]. Forms associated with the cell membrane occur, among others, in cells such as T and B lymphocytes, Treg lymphocytes, NK cells and APC cells [356]. In turn, the soluble molecule PD-L1 is detected in plasma as well as in body fluids, such as pleural effusion of lung cancer patients [357].

In a properly functioning body, after the elimination of pathogens or abnormal infected cells, the PD-1/PD-L pathway has a suppressive effect on T lymphocytes, causing their exhaustion and apoptosis, which prevents autoimmunity. Unfortunately, this natural immunosuppression is used by cancer cells to evade surveillance by the immune system [356].

Therefore, monoclonal antibodies directed against PD-1/PD-L1 are currently used as one of the forms of therapy and treatment of cancer, including lung cancer [352].

In clinical trials, the effectiveness of various models of therapy have been observed, in which preparations such as pembrolizumab, nivolumab, atezolizumab and durvalumab have been used [357].

In clinical trials in the first-line treatment of advanced NSCLC, nivolumab demonstrated an improvement in overall survival outcomes for advanced squamous NSCLC compared to docetaxel (a cytotoxic anticancer drug used in cancer chemotherapy). Similar observations were also obtained but with better results in relation to docetaxelem. In the case of Atezolizumab, its positive impact on patient outcomes was also assessed. The obtained results indicated a beneficial therapeutic path using PD-1/PD-L1 antibodies in patients with advanced NSCLC. In addition, such therapy has a more body-friendly safety profile compared to chemotherapy [358].

##### CTLA4 Pathway

The CTLA-4 molecule, a member of the immunoglobulin superfamily, was expressed primarily by activated T cells. It is an important surface protein and co-inhibitor, localized to activated CD4+/CD8+ T cells to reduce the activity of these cells by binding CD80/CD86/CD28 molecules [359,360].

One of the main tasks of CTLA-4 is to inhibit the function of CD28, a co-stimulatory receptor for T cells. Previous studies suggested that CTLA-4 expression increases upon activation of T cells, while CD28 appears mainly on the surface of naive T cells. Therefore, this molecule seems to have a key role in the regulation of activated T cells, as the literature reports that a lack of CTLA-4 causes unregulated T-cell proliferation [360].

In turn, the deletion or inhibition of T-cell-specific CTLA-4 reduces their ability to control both antitumor and autoimmune responses. Therefore, it is suggested that in the treatment of tumors with a high percentage of T cells that respond to tumor antigens, inhibition of CTLA-4 may be beneficial by restoring anti-tumor immunity by controlling overactive T cells [360].

Currently, as the first-line treatment for lung cancers, such as inoperable malignant pleural mesothelioma (MPM) or NSCLC (with PD-L1 expression in the tumor ≥1% and no EGFR/ALK aberration), treatment with nivolumab and ipilimumab (CTLA-4 inhibitor) is proposed. These formulations, when injected intravenously, have produced favorable treatment results for patients. At the same time, blocking the CTLA-4 molecule using ipilimumab may cause side effects such as colitis and enteritis [361]. Therefore, it is important to find optimal treatments with checkpoint inhibitors so that the benefits outweigh the negative effects. At the same time, current clinical data and basic research give hope for the development of an effective anticancer therapy based on checkpoint inhibitors, because their use can shift the immune balance towards promoting tumor killing and enhancing the immune attack on pathological cells [359].

**Table 4:** Immune checkpoints considered in lung cancer research.

Pathway	Molecules Found on Cancer Cells				Molecules Found on T Lymphocytes				
	Name	Gene and Location	Amino Acid Length and Molecular Weight	Function	Name	Gene and Location	Amino acid Length and Molecular Weight	Function	References
Inhibitory pathway	A2aR	*ADORA2A,* 22q11.23	412 aa; 44.707 kDa	Plays a regulatory role in the adaptive immune system; Extracellular adenosine binds to the receptor, causing a G protein-coupled response, resulting in upregulation of TGF-β and PD-1; Interactions with FOXP3 stimulate CD4+ T cells further suppressing the immune response.	No data				[362,363,364,365]
	VISTA	*C10orf54*, 10q22.1	311 aa, 33.908 kDa	Belongs to the B7 family; Its transcription is partially controlled by p53; Produced in large amounts in tumor-infiltrating lymphocytes, such as myeloid-derived suppressor cells and regulatory T cells.	No data				[364,366,367]
	B7-H3	*CD276*, 15q24.1	534 aa, 57.235 kDa	In benign tissues, it mainly plays an inhibitory role in adaptive immunity by inhibiting T-cell activation and proliferation; In malignant tissues, it inhibits immune responses specific to the tumor antigen; Has non-immune prone-forming functions, such as promotion of migration, invasion, angiogenesis and chemoresistance.	No data				[368,369,370,371]
	PD-L1	*CD274*, 9p24.1	290 aa, 33.275 kDa	Plays a major role in suppressing the adaptive response of the immune system; The binding of PD-L1 to the inhibitory checkpoint molecule PD-1 transmits an inhibitory signal based on interaction with phosphatases (SHP-1 or SHP-2) via the immunoreceptor tyrosine switching motif (ITSM). It reduces proliferation of antigen-specific T cells in lymph nodes while reducing apoptosis in regulatory T cells.	PD-1	*PDCD1*, 2q37.3	288 aa, 31.647 kDa	Protein on the surface of T and B lymphocytes that plays a role in regulating the immune response and promoting self-tolerance by suppressing the inflammatory activity of T lymphocytes; It prevents autoimmune diseases, but it can also prevent the immune system from killing cancer cells.	[346,372,373,374,375,376,377,378]
	PD-L2	PDCD1LG2, 9p24.1	273, 30,957						
	CD80	*CD80*, 13q13.3-q21	288 aa, 33.048 kDa	Can be expressed on antigen presenting cells (APCs) or tumor cells; Interacts with both co-stimulatory (CD28) and coinhibitory receptors (cytotoxic T lymphocytes, antigen 4 (CTLA-4)) and regulates the immune response; The low level of CD80 expression serves as a tumor escape mechanism due to the higher affinity and therefore preferential binding of CTLA-4 to CD80 compared to CD28; In contrast, CD80 overexpression promotes T cell activation and tumor rejection, and CD80 deficiency also increases the immunogenicity of tumor cells.	CTLA-4	*CTLA-4*, 2q33.2	223 aa, 24.656 kDa	Expressed by activated T cells and transmits an inhibitory signal to T cells; CTLA4 expression has been found in many cancers, including non-small-cell lung cancer (NSCLC) tissues and cells; its function in cancer cells is unknown.	[379,380,381,382,383,384,385,386]
	CD86	*CD86*, 3q21	323 aa, 37.021 kDa	Acts in parallel with CD80 (B7-1) as a natural ligand for CD28 and CTLA-4; Promotes T-cell proliferation, function and survival by interacting with CD28 as a co-stimulator, while in activated T cells it interacts with CTLA-4 and acts as a suppressor.					
	Galectin 9	*LGALS9*, 17q11.2	355 aa, 39.518 kDa	HAVCR2/galectin-9 interaction attenuated T-cell expansion and effector function in the tumor microenvironment and chronic infections; Contributes to the formation of tumors through the transformation of cancer cells, regulation of the cell cycle, angiogenesis and cell adhesion.	TIM3	*HAVCR2*, 5q33.3,	301 aa, 33.394 kDa	Belongs to the cell surface receptor proteins of the TIM family; Transmembrane protein of T lymphocytes (CD4+ and CD8+ T cells), myeloid cells (monocytes, macrophages, DCs, mast cells, NK cells) or various cells in various types of cancers; Mediates depletion of CD8+ T cells for proliferation and secretion of cytokines such as TNF-alpha, IFN-gamma and IL-2.	[387,388,389,390,391,392]
	HVEM	*TNFRSF14*, 1p36.32	283 aa, 30.392 kDa	Belongs to the superfamily of TNF receptors; Cytoplasmic region of this receptor binds to several members of the TNF receptor-associated factor (TRAF) family, which may mediate signal transduction pathways that activate the immune response; HVEM overexpression in NSCLC patients was significant in patients with N2 lymph node metastases; Shows a negative correlation with PD-L1 expression in NSCLC patients.	BTLA	*BTLA*, 3q13.2	289 aa, 32.834 kDa	Expressed in lymph nodes, thymus and spleen; Through ITIM, it recruits SHP-1 or SHP-2, which act as phosphatases and dephosphorylate tyrosine, which inhibits immune activation; Has a Grb-2 recognition motif which, when recognized by the Grb-2 protein, promotes PI3K activation and additionally mediates cell proliferation and survival; High BTLA expression may predict poor prognosis in NSCLC patients.	[393,394,395,396,397]
	MHC II	*HLA-DP, -DQ and –DR*, chromosome 6	-	A complex of proteins responsible for the presentation of antigens to T lymphocytes; Correlates with the prognosis of patients with neoplastic diseases and is important in the selection of patients for immunotherapy.	LAG3	*LAG3*, 12p13.31	525 aa,57.449 kDa	Expressed on activated T and B cells, NK cells, and plasmacytoid dendritic cells; Binds to MHC class II with greater affinity than CD4. Negatively regulates cell proliferation, activation and homeostasis of T cells in a similar way to CTLA-4 and PD-1.	[398,399,400,401,402]
Stimulatory pathway	OX40L	*TNFSF4*, 1q25.1	183 aa, 21.050 kDa	Expressed on DC2, macrophages and activated B cells; OX40-OX40L ligation is a source of survival signal for T cells and enables the development of memory T cells; Signaling by these two molecules also polarizes towards a Th2 immune response, even in an environment with low levels of the cytokine IL-4.	OX40	*TNFRSF4*, 1p36.33	277 aa, 29.341 kDa	Present on the surface of activated T cells (mainly CD4+ T cells), but also on NK cells, NKT cells and neutrophils; OX40 plays a key role in NSCLC and SCLC.	[403,404,405,406]
	CD40	*TNFRSF5*, 20q12-q13.2	117 aa,13.158 kDa	Protein found on the surface of antigen-presenting cells, necessary for their activation and providing a co-stimulatory signal to T cells expressing the CD154 molecule; Binding of CD154 to CD40 triggers signaling pathways based on TRAF proteins.	CD40L	*CD40LG*, Xq26	261 aa, 29.274 kDa	Protein found primarily on T lymphocytes, belonging to the TNF superfamily. Its main function is to bind the CD40 molecule on antigen-presenting cells and deliver the co-stimulatory signal required for lymphocyte activation.	[407,408,409,410]
	B7RP1	*ICOSG*, 21q22.3	302 aa, 33.349 kDa	Belongs to the B7 family of co-stimulatory ligands; Shares 19–20% sequence identity with CD80 and CD86; Causes activation and proliferation of T cells; The ICOS/ICOSLG axis promotes antitumor T-cell responses (when activated in Th1 and other Teffs) or protumor responses when triggered in Tregs.	ICOS	*ICOS*, 2q33.2	199 aa, 22.625 kDa	Costimulatory molecule of the CD28 superfamily that is expressed on activated T cells.	[411,412,413,414,415]
	CD70	*CD70*, 19p13.3	193 aa, 21.118 kDa	Belongs to the tumor necrosis factor (TNF) family of ligands; Surface antigen on activated but not resting T and B lymphocytes; Responsible for inducing the proliferation of co-stimulated T cells, increasing the production of cytolytic T cells and contributes to the activation of T cells.	CD27	*CD27*, 12p13.31	260 aa, 29.137 kDa	Necessary for the production and long-term maintenance of T-lymphocyte immunity; Its binding to CD70 activates the signaling cascade leading to differentiation and clonal expansion of T cells; Results in improved survival and memory of cytotoxic T cells and increased production of some cytokines; Transmits signals that lead to the activation of NF-κB and MAPK8 / JNK	[416,417,418,419,420,421]
	GITRL	*TNFSF18*, 1q25.1	177 aa, 20.308 kDa	Regulates T cell responses; May act as a co-stimulator and lower the threshold for T cell activation and T cell proliferation Important for the interaction between activated T cells and endothelial cells; Mediates the activation of NF-kappa-B.	GITR	*TNFRSF18*, 1p36.33	241 aa, 26.000 kDa	Constitutively expressed on CD25+ CD4+ regulatory T cells and is upregulated in all subsets of T cells upon activation; Signaling may promote anticancer and anti-infective immune responses, but may also be a driver of autoimmune diseases.	[422,423,424,425]
	4-1 BBL	*TNFSF9*, 19p13.3	254 aa, 26.625 kDa	Interaction between 4-1BB and 4-1BBL provides co-stimulating signals for various T cells that can be used to discover cancer immunotherapy.	4-1 BB	*TNFRSF9*, 1p36.23	255 aa, 27.899 kDa	Expressed by activated T cells of both CD4+ and CD8+ lines; Increased T cell proliferation, IL-2 secretion, survival and cytolytic activity.	[426,427,428,429,430]
	CD155	*PVR*, 19q13.31	417 aa, 45.303 k Da	Type I transmembrane glycoprotein of the immunoglobulin superfamily; Commonly known as the poliovirus receptor (PVR); It mediates NK cell adhesion and triggers NK cell effector functions	TIGIT	*TIGIT*, 3q13.31	244 aa, 26.319 kDa	Binding to CD155 results in increased secretion of IL10 and decreased secretion of IL12B and inhibition of T cell activation by promoting the production of mature immunoregulatory dendritic cells	[431,432,433,434]

Abbreviations: A2aR—adenosine A2A receptor; VISTA—V-domain Ig suppressor of T-cell activation; B7-H3—B7 homolog 3 protein; PD-1—programmed death receptor-1; PD-L1—programmed death-ligand 1; PD-L2—programmed death-ligand 2; CD80—cluster of differentiation 80; CD86—cluster of differentiation 86; TIM-3—T-cell immunoglobulin mucin-3; HVEM—herpes virus entry mediator; BTLA—B and T lymphocyte attenuator; MHC—major histocompatibility complex; LAG3—lymphocyte activating 3; OX40L—OX40 ligand; CD40—cluster of differentiation 40; CD40L—cluster of differentiation 40 ligand; ICOSLG—inducible T-cell costimulator ligand; ICOS (B7RP-1)—inducible T-Cell costimulator; CD70—cluster of differentiation 70; CD27—cluster of differentiation 27; GITR—glucocorticoid-induced tumor necrosis factor receptor-related protein; GITRL—glucocorticoid-induced tumor necrosis factor ligand-related protein; CD155—cluster of differentiation 155; TIGIT—T-cell immunoreceptor with Ig and ITIM domains.

## 3. Materials and Methods

### 3.1. Search Strategy

**Figure 6:**
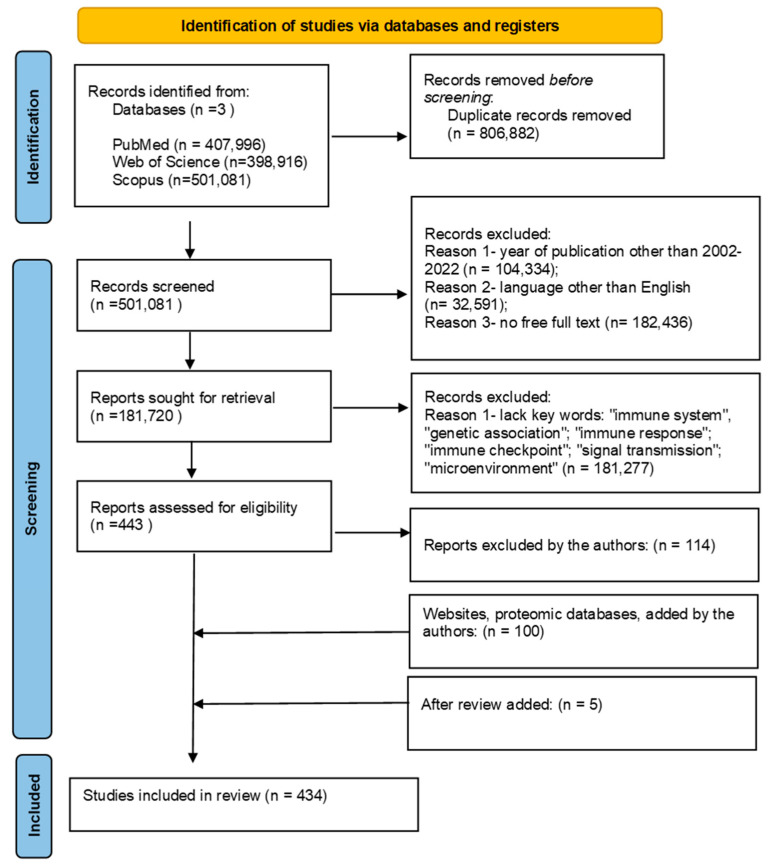
Strategy for searching articles for literature review based on PRISMA flow diagram (based on https://www.prisma-statement.org/PRISMAStatement, accessed on 22 November 2022).

This study was performed on the basis of a meta-analysis of observational studies in accordance with the epidemiological guidelines and the PRISMA statement. To maximize sensitivity, a broad search strategy was driven using the international databases Web of Science, Scopus and PubMed/MEDLINE. The search strategy covered publications from 2002–2022 and used the following keywords: “immune system”; “immune response”; “genetic association”; tumor microenvironment”; “signal transduction”, “immune checkpoint”. Only articles in English were included in the analysis. After removing duplicates, the abstracts of the publications were reviewed by two independent researchers in accordance with the PRISMA abstract list. All disputes were resolved by a third investigator. The detailed search strategy is shown in Figure 6.

### 3.2. Assessment of (Quality) Bias Risk and Data Synthesis Strategy

The remaining articles were then reviewed according to the PRISMA statement. They were then systematically reviewed and assessed against the inclusion and exclusion criteria. All steps were carried out by two independent investigators, and disputes were resolved through discussion with the third investigator.

## 4. Conclusions

The aim of our literature review was to present the importance of immune system disorders and the accompanying changes at the level of cell signaling in the pathogenesis of lung cancer. The information presented by us indicates one of the causes of lung cancer development, which are changes in the tumor microenvironment. Moreover, in our review, we indicated the relationship between deregulation of the immune system and disturbances in cell signaling pathways that contribute to the multistage and multifaceted carcinogenesis of this type of cancer. We hope that the data presented by us will attract the attention of researchers and clinicians and will allow the development of more effective therapies. Despite the numerous scientific studies available in the literature on the etiopathogenesis of various types of lung cancer, it seems important for scientists to undertake an interdisciplinary and multifaceted approach, combining molecular, immunohistochemical and histological methods in order to expand the existing knowledge, which will contribute to faster and more effective diagnosis in the early stages of the disease.

## Figures and Tables

**Table 1:** List of the 20 most mutated genes by lung cancer subtype (adapted from [37]).

AC	LCC	NCSLC	SCLC	ADC
EGFR (31%)	TP53 (68%)	EGFR (25%)	TP53 (63%)	TP53 (57%)
KRAS (17%)	KRAS (22%)	KRAS (16%)	RB1 (46%)	EGFR (7%)
TP53 (42%)	EGFR (5%)	TP53 (31%)	LRP1B (25%)	LRP1B (37%)
STK11 (11%)	STK11 (8%)	ARID1A (25%)	KMT2D (16%)	PIK3CA (8%)
BRAF (3%)	PIK3CA (4%)	PIK3CA (4%)	EGFR (6%)	NFE2L2 (12%)
KEAP1 (15%)	NRAS (3%)	ALK (17%)	NOTCH1 (9%)	KRAS (4%)
MET (3%)	MET (3%)	BRAF (2%)	PIK3CA (7%)	KMT2D (17%)
LRP1B (28%)	KEAP1 (21%)	MET (2%)	PTEN (6%)	CDKN2A (11%)
ERBB2 (2%)	AR (32%)	STK11 (7%)	EP300 (8%)	FAT4 (16%)
PIK3CA (4%)	BRAF (2%)	KEAP1 (16%)	FAT1 (9%)	KMT2C (15%)
SMARCA4 (9%)	PTEN (4%)	CDKN2A (4%)	ATRX (8%)	KEAP1 (9%)
NF1 (8%)	CDKN2A (5%)	ATM (13%)	KMT2C (8%)	NF1 (11%)
ATM (7%)	LRP1B (28%)	KMT2D (24%)	CREBBP (8%)	PTEN (7%)
FAT1 (8%)	POLQ (28%)	SMARCA4 (10%)	GRIN2A (8%)	ERBB4 (8%)
PTPRT (9%)	HRAS (4%)	NF1 (9%)	FAT4 (10%)	FAT1 (12%)
KMT2C (9%)	GNAS (4%)	SETBP1 (10%)	ROS1 (8%)	ROS1 (10%)
RBM10 (8%)	ERBB4 (5%)	FAT1 (15%)	ERBB4 (6%)	PREX2 (13%)
KMT2D (7%)	ATM (6%)	ERBB2 (2%)	ARID1A (6%)	PDE4DIP (11%)
FAT4 (16%)	FLT3 (7%)	TET2 (8%)	ZNF521 (7%)	ZFHX3 (9%)
ERBB4 (5%)	ZFHX3 (21%)	NOTCH1 (3%)	MTOR (5%)	GRIN2A (10%)

Abbreviations: EGF—epidermal growth factor receptor; KRAS—Kirsten rat sarcoma virus; TP53—tumor protein p53; STK11—serine/threonine kinase 11; BRAF—B-Raf proto-oncogene, serine/threonine kinase; Keap1-Kelch-like ECH-associated protein 1; MET—MET proto-oncogene, receptor tyrosine kinase; LRP1B—LDL receptor-related protein 1B; ERBB2-Erb-B2 receptor tyrosine kinase 2; PIK3CA—phosphatidylinositol-4,5-bisphosphate 3-kinase catalytic subunit alpha; SMARCA4—SWI/SNF-related, matrix-associated, actin-dependent regulator of chromatin, subfamily A, member 4; NF1—neurofibromin 1; ATM-ATM serine/threonine kinase; FAT1—FAT atypical cadherin 1; PTPRT-protein tyrosine phosphatase receptor type T; KMT2C-lysine methyltransferase 2C; RBM10 (RNA binding motif protein 10; KMT2D—lysine methyltransferase 2D; FAT4—FAT atypical cadherin 4; ERBB4—Erb-B2 receptor tyrosine kinase 4; NRAS—NRAS proto-oncogene, GTPase; AR—androgen receptor; PTEN—phosphatase and tensin homolog; CDKN2A—cyclin-dependent kinase inhibitor 2A; POLQ—DNA polymerase theta; HRAS—HRas proto-oncogene, GTPase; GNAS—GNAS complex locus; FLT3—Fms-related receptor tyrosine kinase 3; ZFHX3—zinc finger homeobox 3; ARID1A—AT-rich interaction domain 1A; ALK—ALK receptor tyrosine kinase; SETBP1—SET binding protein 1; TET2—tet methylcytosine dioxygenase 2; Notch 1—neurogenic locus notch homolog protein 1; RB1—RB transcriptional corepressor 1; EP300 Gene—E1A binding protein P300; ATRX—ATRX chromatin remodeler; CREBBP—CREB binding protein; GRIN2A—glutamate ionotropic receptor NMDA type subunit 2A; ROS1—ROS proto-oncogene 1, receptor tyrosine kinase; ZNF521—zinc finger protein 521; MTOR—mechanistic target of rapamycin kinase; NFE2L2—NFE2-like BZIP transcription factor 2; REX2—phosphatidylinositol-3,4,5-trisphosphate-dependent Rac exchange factor 2; PDE4DIP—phosphodiesterase 4D interacting protein.

**Table 2:** Disturbances in cell signaling pathways involved in the development and progression of lung cancer.

Type	Gene and Location	Protein Name	Amino Acid Length	Molar Weight [kDa]	Function	Function in Lung Cancer	References
Cell signaling pathway stimulating cell growth	*EGFR,* 7p11.2	EGFR	1210	134.277	The main regulator of the cell proliferation process; Responsible for endocrine and paracrine regulation of cell growth and proliferation.	Acts as a strong oncogene in the case of deregulatory changes; Its dysregulation has been observed in 40% of NSCLC and ADC cases; Its increased expression is associated with a lower prognosis of patients.	[55,56,57,58,59]
	*BRAF*, 7q34	BRAF	766	84.437	A serine/threonine kinase involved in the transduction of cellular signals related to growth; Plays a key role in the regulation of MAPK/ERK signaling.	Mutations are detected in about 3% of lung cancer cases; It is a proto-oncogene; The V600 mutation in loop A is the most common type of mutation; A mutation is incompatible with RET, Ras mutations in lung cancer.	[60,61,62]
	*ERBB2*, 17q12	HER2	1255	137.910	It is a membrane-associated tyrosine kinase and is responsible for regulating cell proliferation.	Its mutation occurs in non-smokers; The mutation affects about 2% of lung cancer cases; It is an oncogene.	[63,64,65]
	*KRAS,* 12p12.1	KRAS	189	21.656	Part of the signaling of the RAS/MAPK pathway; Responsible for cell proliferation and differentiation.	It is a proto-oncogene; Mutation involved in the pathogenesis of lung adenoma; Mutations are more common in smokers and men.	[66,67,68,69]
	*PIK3CA,* 3q25	PIK3CA	1068	124.284	It is a kinase involved in a number of cellular functions such as growth, proliferation, differentiation, motility and secretion.	One of the most frequently mutated onco-genes along with KRAS; Relatively little information about its mutation in NSCLC; Lack of complete studies on the relationship of its amplification in lung cancer (SCLC).	[70,71,72]
	*ALK*, 2p32.2	ALK	1620	176.442	Plays a role in RAS activation; Participates in cellular communication and the proper development and functioning of the nervous system.	Occurs in 7% of NSCLC cases; Negative for KRAS or EGFR mutations; Mutations favor the development of ADCs; It is associated with a tendency to metastasize to the pleura and pericardium; Mutations are seen more often in women.	[73,74]
	*TITF1*, 14q13.3	TITF1	371	38.596	The main transcription factor necessary for the development of peripheral airways	It is a specific marker of ADC development; Can also be expressed in SCLC.	[75,76,77]
	*c-MYC*, 8q24.21	MYC	439	48.804	It is a transcription factor; Participates in cell proliferation and differentiation.	It is a proto-oncogene; *l-* and *n-MYC* amplification is specific to SCLC and LCNEC development.	[78,79,80]
	*l-MYC*, 1p34.2	MYC	364	40.327			[80,81]
	*n-MYC*, 2p24.3	MYC	464	49.561			[79,80,82]
Szlak genów supresorowych guza	*TP53*, 17p13	P53	393	43.653	Cell-cycle transcription factor; It is a kind of cellular guardian, protecting against instability and genetic abnormalities; It is also a sensor of stress signals, e.g., DNA damage, the activation of oncogenes; It is a tumor suppressor; Regulated by Mdm2 and p14ARF.	It is the most frequently mutated gene in lung cancer: 90% of SCLC and LCNEC and 50% of NSCLC; The mutation is closely related to exposure to carcinogens, especially smoking and benzopyrene; Mutations in regulatory genes: 65 cases of NSCLC were related to Mad2; the mutation in p14ARF affects SCLC, LCNEC and ADC.	[83,84,85]
	*RB1*, 13q14.2	Rb1	928	106.159	Tumor suppressor gene; It is an effector of p53-mediated G1/s cycle arrest via activation of p21 kinase inhibitor.	The lack of Rb protein is one of the most common escape mechanisms from the G1 checkpoint seen in SCLC; in NSCLC, hyperphosphorylation of Rb occurs; Loss of Rb occurs in 70% of LCNEC cases, 90% of SCLC cases and 15% of NSCLC cases.	[86,87,88]
	*STK11*, 19p13.3	STK11	433	48.636	It is a serine/threonine kinase; Acts as a tumor suppressor; Is the precursor kinase to AMPK, an essential component of cellular metabolism and the maintenance of energy homeostasis.	A mutation of this gene has been observed in ADC; It is a critical barrier to new lung formation, controls initiation, differentiation and metastasis; Typically, mutations are in smokers and are associated with a mutation in KRAS.	[89,90,91]
Szlak związany z unikaniem apoptozy	*Bax*, 19q13.33	Bax	192	21.184	Bcl-2 and Bax are key factors of mitochondrial apoptosis involved in the control of outer membrane permeabilization, resulting in the release of cytochrome C.	Bax heterodimerizes with Bcl-2 to control the level of susceptibility to apoptosis; Bcl-2 is overexpressed in SCLC and LCNEC and downregulated in NSCLC; Bax is downregulated in SCLC and upregulated in NSCLC; Abnormalities in the Bcl-2:Bax ratio are observed in 95% of SCLC cases and 25% of NSCLC cases.	[92,93,94,95,96]
	*Bcl-2*, 18q21.33	Bcl-2	239	26.266			[93,94,96,97,98]
	*FASL*, 1q24.3	TNFL6	281	31.485	Its interaction with the Fas transmembrane receptor (FasR) on target cells induces the course of apoptosis; Has immunoregulatory importance and is involved in the process of carcinogenesis.	Like the FasR receptor, FasL has been shown to be downregulated in 70% of NSCLC cases; In SCLC, there is no expression of FasR but high expression of FasL (50% of cases).	[99,100,101]
	*E2F1*, 20q11.22	E2F1	437	46.920	It is a transcription factor involved in the control of the G1/S cell cycle; Can mediate both p53-dependent and non-p53-dependent proliferation and apoptosis.	Low levels of expression are detected in NSCLC and have been suggested to increase cancer cell survival; High levels of expression are seen in SCLC and increase tumor cell proliferation.	[102]
	*TERT*, 5p15.33	TERT	1132	126.997	Responsible for maintaining telomere ends; It is involved in the aging process of cells.	High expression of telomerase contributes to the early stages of immortalization of cancer cells; Elevated telomerase levels are observed in 80% of NSCLC cases and nearly 100% of SCLC cases; Telomerase is expressed in checkpoint-deficient cancer cells via p53 or Rb.	[103,104,105]

Abbreviations: EGFR—epidermal growth factor receptor; BRAF—serine/threonine-protein kinase B-raf; MAP/ERK—mitogen-activated protein kinase/extracellular signal-regulated kinase ½; ERBB2—receptor tyrosine-protein kinase erbB-2; HER2—human epidermal growth factor receptor 2; RET—ret proto-oncogene; Ras—rat sarcoma virus; KRAS—Kirsten rat sarcoma virus; PIK3CA—phosphatidylinositol-4,5-bisphosphate 3-kinase catalytic subunit alpha; ALK—tyrosine kinase receptor; TITF1—thyroid transcription factor 1; MYC—Myc proto-oncogene protein; TP53—cellular tumor antigen p53; Mdm2—mouse double minute 2 homolog MDM2; p14ARF—ARF tumor suppressor; RB1—retinoblastoma-associated protein; STK11—serine/threonine-protein kinase STK11; AMPK—5’AMP-activated protein kinase; BAX—apoptosis regulator BAX; BCL2—apoptosis regulator Bcl-2; TNFL6—tumor necrosis factor ligand superfamily member 6; E2F1—E2F transcription Factor 1; E2F1—transcription factor E2F1; TERT—telomerase reverse transcriptase.

**Table 3:** miRNAs involved in the pathogenesis of lung cancer.

miRNA	Genetic Location	Target	Mechasims	References
miR-7	miR-7 is derived from three precursors: pri-miR-7-1 (9q21), pri-miR-7-2 (15q26) and andpri-miR-7-3 (19q13)	EGFR	Sustaining proliferative signaling	[139,140]
miR-27	19p13.12	EGFR	Sustaining proliferative signaling	[141,142,143,144]
miR-30	6q13	EGFR	Sustaining proliferative signaling	[145,146,147]
miR-34	1p36.22	EGFR	Sustaining proliferative signaling	[148,149,150]
		PD-L1	Avoiding immune destruction and tumors promoting inflammation	
miR-128	2q21.3	EGFR	Sustaining proliferative signaling	[151,152,153,154]
		VEGF	Inducing angiogenesis	
miR-134	14q32.31	EGFR	Sustaining proliferative signaling	[155,156,157]
miR-542	Xq26.3	EGFR	Sustaining proliferative signaling	[158,159,160]
miR-1258	2q31.3	Grb2	Sustaining proliferative signaling	[161,162,163]
miR-148a	7p15.2	SOS	Sustaining proliferative signaling	[164,165,166]
miR-181a	chromosome 1 (37.p5)	RAS	Sustaining proliferative signaling	[167,168]
miR-193a	17q11.2	RAS	Sustaining proliferative signaling	[169,170,171]
miR-760	1p22.1	ROS1	Sustaining proliferative signaling	[172,173,174]
miR-96	7q32.2	EML4-ALK	Sustaining proliferative signaling	[175,176,177]
miR-200c/	12p13.31	PI3K	Sustaining proliferative signaling	[178,179,180,181,182]
	chromosome 1	ZEB1/2	Activating invasion and metastasis	
miR-200		VEGF	Inducing angiogenesis	
		PD-L1	Avoiding immune destruction and tumors promoting inflammation	
miR-520a	19q13.42	PI3K	Sustaining proliferative signaling	[183,184,185]
miR-641	19q13.2	MDM2	Evading growth suppressors	[186,187,188]
miR-660	Xp11.23	MDM2	Evading growth suppressors	[189,190]
miR-15a	13q14.2	CDK/Cyclin	Evading growth suppressors	[191,192,193]
miR-16	13q14	CDK/Cyclin	Evading growth suppressors	[194,195,196,197]
		Bcl	Resisting cell death	
miR-449a	5q11.2	E2F	Evading growth suppressors	[198,199,200,201]
miR-299	14q32.31	hTERT	Enabling replicative immortality	[202,203,204]
miR-491	9p21.3	hTERT	Enabling replicative immortality	[205,206,207]
miR-512	19q13.42	hTERT	Enabling replicative immortality	[208,209,210]
miR-29	7q32.3	DNMT	Enabling replicative immortality	[211,212,213]
miR-146A	5q33.3	Smad	Activating invasion and metastasis	[214,215,216]
miR-21	17q23.1	TGF-β	Activating invasion and metastasis	[217,218,219,220,221]
		PTEN	Avoiding immune destruction and Tumors promoting inflammation	
miR-23	no data	TGF-β	Activating invasion and metastasis	[222,223,224]
miR-99A	21q21.1	TGF-β	Activating invasion and metastasis	[225,226,227]
miR-155	21q21.3	TGF-β	Activating invasion and metastasis	[228,229,230,231]
miR-192	11q13.1	TGF-β	Activating invasion and metastasis	[232,233,234,235]
miR-115	no data	TGF-β	Activating invasion and metastasis	[236,237]
miR-520	19q13.42	TGF-β	Activating invasion and metastasis	[238,239,240]
miR-497	17p13.1	HDGF	Inducing angiogenesis	[241,242,243,244]
miR-494	14q32.31	PTEN	Inducing angiogenesis	[245,246,247,248]
miR-126	9q34.3	VEGF	Inducing angiogenesis	[249,250,251,252]
miR-33b	17p11.2	LDHA	Deregulating cellular energetics	[253,254,255]
miR-144	17q11.2	GLUT	Deregulating cellular energetics	[256,257,258]
miR-124	8p23.1	Akt	Deregulating cellular energetics	[259,260,261]
miR-199a	19p13.2	HIF-1	Deregulating cellular energetics	[262,263,264]
miR-31-5p	9p21.3	FIH	Deregulating cellular energetics	[265,266,267]
miR-28	3q28	PD-1PTEN	Avoiding immune destruction and tumors promoting inflammation	[268,269,270]
miR-138	16q13	PD-1	Avoiding immune destruction and tumors promoting inflammation	[271,272]
		PD-L1		
miR-197	1p13.3	CKS1B	Avoiding immune destruction and tumors promoting inflammation	[273,274,275]
miR-513	Xq27.3	PD-L1	Avoiding immune destruction and tumors promoting inflammation	[276,277,278,279]
miR-20	13q31.3	PTEN	Avoiding immune destruction and tumors promoting inflammation	[280,281,282]
miR-130	11q12.1	PTEN	Avoiding immune destruction and tumors promoting inflammation	[283,284,285]
miR-424	Xq26.3	CD80	Avoiding immune destruction and tumors promoting inflammation	[286,287,288,289]
miR-136	14q32.2	CTLA-4	Avoiding immune destruction and tumors promoting inflammation	[290,291,292,293]
miR-301b	22q11.21	BH3	Resisting cell death	[294,295,296,297,298]

Abbreviations: EGFR—epidermal growth factor receptor; PD-L1—programmed death-ligand 1; VEGFA—vascular endothelial growth factor A; Grb2—growth factor receptor bound protein 2; SOS—Ras/Rac guanine nucleotide exchange factor; Ras—rat sarcoma virus; ROS1—ROS proto-oncogene 1, receptor tyrosine kinase; EML4—echinoderm microtubule-associated protein-like 4; ALK—anaplastic lymphoma kinase; PI3K—phosphoinositide 3-kinase; ZEB1 zinc finger E-box binding homeobox 1; MDM2—mouse double minute 2 homolog; CDK—cyclin-dependent kinase; Bcl—B-cell lymphoma; hTERT—telomerase reverse transcriptase; DNMT—DNA methyltransferase; SMAD—suppressor of mothers against decapentaplegic; TGF-β—transforming growth factor beta; PTEN—phosphatase and tensin homolog; HDGF—heparin binding growth factor; LDHA—lactate dehydrogenase A; GLUT—glucose transporter; HIF-1—hypoxia inducible factor 1 subunit alpha; FIH—hypoxia-inducible factor inhibitor; CKS1B—CDC28 protein kinase regulatory subunit 1B; CD80—cluster of differentiation 80; CTLA-4—cytotoxic T cell antigen 4; BH3—interacting domain death agonist.

## Data Availability

Not applicable.
